# The High Cost of Living

**Published:** 1914-05

**Authors:** 


### The High Cost of Living

As this matter is one of very general interest and not unconnected with either public health or the economies of the medical profession, it is allowable to discuss it editorially, especially as we are inclined to an optimistic view which is not the common one.

The necessities of life include clothing, heat, shelter and food. We do not believe that any of these have increased in price within the last twenty-five years, except food. We expect that most of our readers will disagree with this statement, at first thought. Your clothes cost more than formerly, to be sure, but this is inevitable if you wear shirts with valvulæ conniventes all over the front instead of the old-fashioned, simple garment; if you substitute silk for cotton; if you buy your outer garments of a man who, instead of working twelve hours a day to the detriment of his health and starvation of his soul, spends six or eight hours a day in an automobile in the summer and half as much in a white waistcoat and kid gloves in the winter; if you pay fifty cents for an article worth ten or fifteen when it is passed over the counter. But, with due care, even very adequate clothes do not, on the average, necessarily cost more today than a generation ago. Coal is up—to about the price which the writer's grandfather paid for it when it first came on the market as a practical commodity—and natural gas is a trifle higher than when it first entered some of the more fortunate fields, but, on the average, fuel does not cost you as many days' work as it did our predecessors, who hauled it from their own woods and split it and brought it in from the wood shed. Indeed, thanks to improvement in heating apparatus, it costs you very little more to have your house heated all over all the time than it did even your father, who bought coal in a city, to have an intermittent supply of heat. Light may be absolutely more expensive than formerly, for similar reasons, but, for those in the natural gas district, it is cheaper than any comparable means of lighting that man has ever used.

You may pay more for your house than your father or grandfather or your former self, but is it the same house? We do not mean in the literal sense nor do we imply that you are granting yourself greater luxury in the selection of a house or its neighborhood. Taking the same grade of house for the present and a generation ago, the addition merely of a very simple bath room implies an actual expense in interest and up-keep to the owner of at least a dollar a month. The very word *house,* on this and other accounts, means something more than it meant formerly. We contend that the poorest householder saves this dollar a month, not in comfort, but in actual expense, for sickness. Economically, a house consists in an accumulation of human labor —even material really involves labor in the ultimate analysis. The house of today costs at least 50 per cent. more in labor than that of a generation ago. Its rental is not proportionately greater. In another sense, a house is invested capital. A while ago we came across an old family letter in which it was mentioned that the returns from a house were only 17 per cent. Today the average gross return is 8-10 per cent. and the net income seldom over 5 per cent. In other words, considering what he gets from either the side of labor or capital, the average householder today is getting his shelter at a marked reduction.

Food is undoubtedly higher in price than formerly, but it is, on the whole, food of better quality carrying with it some degree of insurance against sickness and against groess loss by spoiling. Wheat flour costs almost exactly the same as it did seventy-five years ago. Sugar, tea and coffee have decreased in price. So have water and ice, if we count labor and safety. If one benefits by advance in knowledge, understands what foods he needs, and realizes that many articles that were formerly considered luxuries and scarcely of any food value are not only more pleasing to the palate but actually cheaper, comparing physiologic values with price, and if, in addition, he takes the trouble to buy certain things in small wholesale lots, he can set just as good a table today as twenty-five years ago, for the same cost.

The increase in many prices represents the practical development of democratic ideals. Every direct or fairly direct purchase of labor means a greater expense; in other words, a fairer sharing of aggregate wealth. But most articles produced or manufactured on a large scale are cheaper at a high wage for labor than the same or supplanted articles produced or manufactured on a small scale at a low wage. To take a concrete example, cobblers by advancing their prices commensurate with other laborers have practically driven themselves out of business, but the consumer can buy shoes, throw them away when they get to the stage of patching or soling and buy new ones, at not much if any greater aggregate expense than his father or grandfather underwent for less pleasing footgear. Moreover, labor saving devices have rendered it possible for the average family to dispense with the direct expense for labor, especially domestic labor, which a family of the same means would have been obliged to employ a generation ago.

For the average family, we are inclined to believe that the cost of labor saving devices and the increased price of labor directly employed, are offset by the saving which they entail, not in comfort nor in actual leisure, but in other expenses and in time saved which can be made directly profitable. For example, the two items of hardwood floors and a vacuum cleaner, representing a comparatively slight initial expense, eliminate almost entirely extra wages for house cleaning. They afford, also, the comfort of being free from these periods of domestic chaos, of feeling clean all the time; they eliminate the liability to certain sicknesses from exposure and physical strain, and they allow the use of floor coverings of greater elegance, higher initial cost but of greater durability.

There has never been a time when the average citizen had so many opportunities for self-improvement, recreation and even entertainment at a small cost as at present. But, the temptation to an easier, more intellectual or more pleasurable existence, tends to extravagance in many directions. It is scarcely logical to expect as much prosperity from eight rather easy-going hours of labor as from ten hours under pressure, but this paradox would undoubtedly be achieved if the extra two hours were not occupied with spending money in ways that further reduce the efficiency.

There never was a time when the compulsory or voluntary taxation of the wealthier and more efficient part of the community provided so liberally for the comfort and protection of the poorer or less efficient part. This fact has resulted in increasing the stress of living for a large part of the community. We think it must be recognized that, from the philanthropic standpoint, the community cannot be fairly divided into supporting and assisted or supported portions, but that neither compulsion at the hands of the government nor too great suasion at private hands should compel the middle class to spend more than a very insignificant amount in benevolence which they do not ask for themselves but which, on the other hand, they cannot afford for others except at undue sacrifice. Our taxing authorities, charitable and religious institutions, are all at fault in this particular, and we believe that the enormous sums of money for philanthropic purposes, levied against poor taxpayers, or begged from the same class, and the army of philanthropic executives and workers taken from directly productive occupations, have a good deal to do with the High Cost of Living. The same work should proceed, but with rigid economy, with no waste on the idle and dishonest, and the burden should be carried by those able to bear it.

In the aggregate, the community gets more comfort, for fewer days of less arduous labor, than ever before. The complaint of the H. C. L. amounts largely, therefore, to a complaint that the average man cannot enjoy still greater luxury than he does. The complaint does not even mean, in the relative sense of numbers, that one class is exploited for the benefit of another. It comes most loudly and most numerously from persons whose grandfathers if not whose fathers were, in a sense, exploited for the benefit of a small, favored class, and who bore this exploitation without complaint. And their grandsons, or even sons, in complaining that things and labor cost too much, are virtually demanding that they should be allowed to exploit precisely the same class from which they have emerged.

The possibilities of living have increased enormously within a few years. For example, the great majority of automobiles are owned by persons who, under the conditions of twenty-five years ago, would never have dreamed of owning a carriage, scarcely even a buggy. They spend on this one luxury—we are not speaking now of commercial vehicles or those used in business to facilitate the earning of as much or more money than the automobile costs—more than, under those conditions, would have been spent on all items beyond absolute necessities, with a more rigid line between necessities and luxuries than at present, yet they complain about the H. C. L. Such a complaint implies one of two things, either grudging a commensurate degree of comfort to the hard working class or a demand for more luxury than the world's progress has yet rendered possible.

